# Supplementary material for: The Plasmatic Aldosterone and C-Reactive Protein Levels, and the Severity of Covid-19: The Dyhor-19 Study
Source: J Clin Med. 2020 Jul 21;9(7):2315. doi: 10.3390/jcm9072315 (PMC7408691; doi:10.3390/jcm9072315)
Supplement: Supplementary file 1 [file jcm-09-02315-s001.zip › Supplemental Table 1.docx]

**Supplemental Table 1.** Clinical characteristics of patients with Covid-19 according to disease severity classified in three groups: mild (OS max ≤ 3), moderate (OS max = 4) and severe (OS max ≥ 5).

| Patients characteristics | | Disease SEVERITY | | | | | TOTAl | |
| --- | --- | --- | --- | --- | --- | --- | --- | --- |
|  |  | **Mild**  (N=10) | **Moderate**  (N=19) | | **Severe**  (N=15) | | (N=44) | |
| Age, median (IQR) – yrs | | 60 (55.3-69.3) | 69.5 (57.5-75.8) | | 70 (50.5-75.5) | | 66.5 (53-75.3) | |
| Female sex – n. (%) | | 5 (50) | 4 (21) | | 8 (53) | | 17 (38.6) | |
| Diagnosis of Covid-19 – n (%) | |  |  | |  | |  | |
|  | Positive | 9 (90) | 18 (95) | | 14 (93) | | 41 (93.2) | |
|  | Suspected | 1 (10) | 1 (5) | | 1 (7) | | 3 (6.8) | |
| Coexisting conditions– n (%) | |  |  | |  | |  | |
|  | Hypertension | 5 (50) | 10 (52.6) | | 10 (66.7) | | 25 (56.8) | |
|  | Diabetes mellitus | 4 (40) | 4 (21.1) | | 7 (46.7) | | 15 (34.1) | |
|  | Obesity | 2 (20) | 4 (21.1) | | 5 (33.3) | | 11 (25) | |
|  | Cardiovascular disease | 2 (20) | 8 (42.1) | | 6 (40) | | 16 (36.4) | |
|  | Stroke | 1 (10) | 1 (5.3) | | 3 (20) | | 5 (11.4) | |
|  | Chronic kidney disease (GFR < 60 ml/min) | 1 (10) | 1 (5.3) | | 5 (33.3) | | 7 (15.9) | |
|  | Cancer | 1 (10) | 6 (31.6) | | 1 (6.7) | | 8 (18.2) | |
|  | Dysthyroid disease | 1 (10) | - | | 4 (26.7) | | 5 (11.4) | |
|  | History of organ transplantation | 1 (10) | - | | 3 (20) | | 4 (9.1) | |
| Long-term anti-hypertensive treatment – n (%) | |  |  | |  | |  | |
|  | Angiotensin-converting enzyme inhibitor | 2 (20) | 4 (21.1) | | 4 (26.7) | | 10 (22.7) | |
|  | Angiotensin-receptor blocker | 2 (20) | 3 (15.8) | | 1 (6.7) | | 6 (13.6) | |
|  | Calcium-channel blocker | 0 | 5 (26.3) | | 1 (6.7) | | 6 (13.6) | |
|  | Beta-blocker | 1 (10) | 5 (26.3) | | 8 (53.3) | | 14 (13.8) | |
|  | Thiazide diuretic | 1 (10) | 1 (5.3) | | 1 (6.7) | | 3 (6.8) | |
|  | Anse diuretic | - | 2 (10.5) | | 5 (33.3) | | 7 (15.9) | |
| Signs and symptoms of Covid-19 – n (%) | |  |  | |  | |  | |
|  | Fever | 8 (80) | 19 (100) | | 11 (73.3) | | 38 (86.4) | |
|  | Myalgia | 1 (10) | 8 (42.8) | | 5 (35.7) | | 14 (32.6) | |
|  | Cough | 6 (60) | 14 (73.7) | | 8 (53.3) | | 28 (65.1) | |
|  | Breathlessness | 3 (30) | 12 (63.2) | | 9 (60) | | 24 (54.5) | |
|  | Diarrhea | 1 (10) | 6 (31.6) | | 5 (33.3) | | 12 (27.3) | |
|  | Headache | - | 1 (5.3) | | 1 (6.7) | | 2 (4.5) | |
|  | Anosmia | - | 7 (36.8) | | 1 (6.7) | | 8 (18.2) | |
|  | Dysgeusia | 1 (10) | 6 (31.6) | | 2 (13.3) | | 9 (20.5) | |
| Delay from onset symptoms median (IQR) – day | | 8 (5-10) | 8 (6-10) | | 7 (4-8) | | 8 (5-10) | |
| Care during hospitalization | |  |  | |  | |  | |
|  | Duration mean (SD) – day | 5.5 (4.1) | 13.9 (10.5) | | 18.8 (14.1) | | 13.1 (10.9) | |
|  | Corticosteroid therapy mean (SD) – day | 0.7 (2.2) | | 1.3 (2.8) | | 3.3 (3.8) | | 1.8 (3.1) |
|  | Use of antibiotic agents – n (%) | 5 (50) | | 11 (57.9) | | 14 (93.3) | | 30 (68.2) |
|  | Use of vasoactive drug – n (%) | - | | - | | 7 (46.7) | | 7 (15.91) |
| OS denotes ordinal scale, IQR interquartile range, n number, eGFR estimated glomerular filtration rate. | | | | | | | | |
