# Supplementary figures and images for: The Plasmatic Aldosterone and C-Reactive Protein Levels, and the Severity of Covid-19: The Dyhor-19 Study
Source: J Clin Med. 2020 Jul 21;9(7):2315. doi: 10.3390/jcm9072315 (PMC7408691; doi:10.3390/jcm9072315)

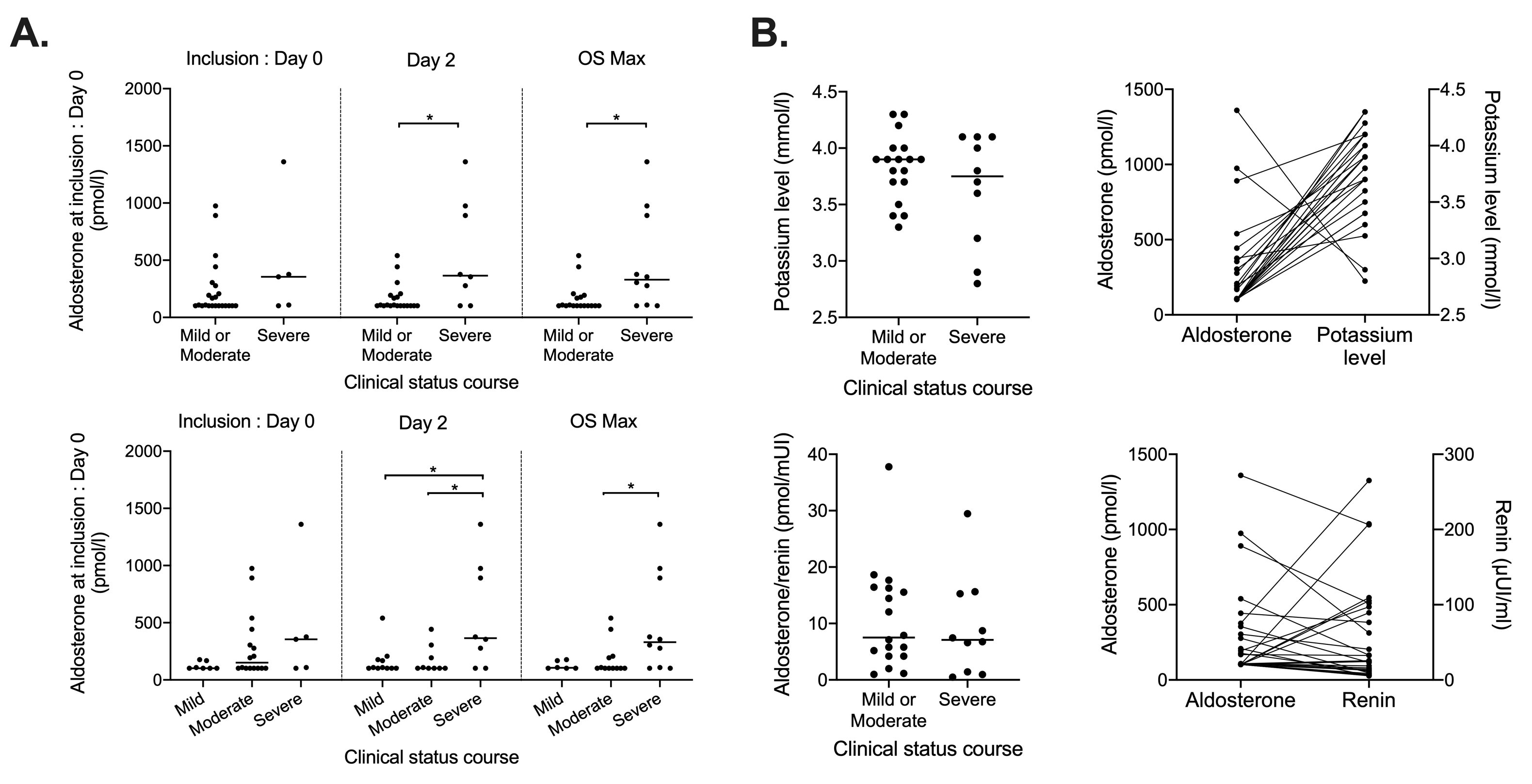

Supplement: Supplementary file 1 [file jcm-09-02315-s001.zip › Supplemental Figure 1.tiff]
